# Bispecific Antibodies: A New Era of Treatment for Multiple Myeloma

**DOI:** 10.3390/jcm9072166

**Published:** 2020-07-09

**Authors:** Xiang Zhou, Hermann Einsele, Sophia Danhof

**Affiliations:** Department of internal medicine II, University Hospital Würzburg, Oberdürrbacher Street 6, D-97080 Würzburg, Germany; Einsele_H@ukw.de (H.E.); danhof_s@ukw.de (S.D.)

**Keywords:** bispecific antibody, multiple myeloma, relapse, refractory, immunotherapy

## Abstract

Despite the introduction of novel agents such as proteasome inhibitors, immunomodulatory drugs, and autologous stem cell transplant, multiple myeloma (MM) largely remains an incurable disease. In recent years, monoclonal antibody-based treatment strategies have been developed to target specific surface antigens on MM cells. Treatment with bispecific antibodies (bsAbs) is an immunotherapeutic strategy that leads to an enhanced interaction between MM cells and immune effector cells, e.g., T-cells and natural killer cells. With the immune synapse built by bsAbs, the elimination of MM cells can be facilitated. To date, bsAbs have demonstrated encouraging results in preclinical studies, and clinical trials evaluating bsAbs in patients with MM are ongoing. Early clinical data show the promising efficacy of bsAbs in relapsed/refractory MM. Together with chimeric antigen receptor-modified (CAR)-T-cells, bsAbs represent a new dimension of precision medicine. In this review, we provide an overview of rationale, current clinical development, resistance mechanisms, and future directions of bsAbs in MM.

## 1. Introduction

Multiple myeloma (MM) is a plasma cell neoplasia, which is often complicated by osteolytic lesions, anemia, renal dysfunction, hypercalcemia, progressive bone marrow dysfunction, infections, and extramedullary manifestations [1,2]. In the past few decades, the introduction of novel agents, including proteasome inhibitors (PIs), immunomodulatory drugs (IMiDs), and high dose chemotherapy with autologous stem cell transplant (SCT), has improved the survival outcome of patients significantly [3]. It has also been reported that first-line allogeneic SCT could lead to long-term disease control in patients with high-risk MM through the graft-versus-myeloma effect [4,5,6]. With these developments in the treatment of MM, the five-year relative survival ratios of patients increased from 28% for 1973–1982 to 41% during 2003–2013 [7]. However, MM largely remains an incurable disease. Most patients suffer from relapse during the course of the disease and develop PI-and/or IMiD-resistance. In particular, the management of late-stage relapsed/refractory (RR) MM still represents a formidable challenge in the clinical practice.

In the last few years, several monoclonal antibodies (mAbs) targeting specific surface antigens on MM cells have been developed for the treatment of RRMM. The first-in-class mAb that has been approved for myeloma treatment is the signaling lymphocyte activation molecule family 7 (SLAMF7)-directed agent elotuzumab [8]. In the randomized, multicenter, phase 3 ELOQUENT-2 trial, RRMM patients who received elotuzumab, lenalidomide, and dexamethasone (ELd) showed a significantly superior progression-free survival (PFS; 19.4 months versus 14.9 months, *p* < 0.001) and a higher overall response rate (ORR; 79%, versus 66%, *p* < 0.001) when compared with the control group of lenalidomide and dexamethasone (Ld) after a median follow-up of 24.5 months [9]. Based on experience from the ELOQUENT-2 trial, elotuzumab obtained U.S. Food and Drug Administration (FDA) approval in November 2015. In addition, daratumumab, a mAb targeting CD38, has also shown promising efficacy in various clinical trials, and may achieve synergistic anti-tumor effects, together with PIs and IMiDs, in patients with RRMM [10,11,12,13]. Other than elotuzumab, which is characterized by a lack of single-agent activity, daratumumab monotherapy has been shown to be effective even in patients with refractory myeloma [10]. Moreover, daratumumab containing combination regimens have been approved for first-line treatment, e.g., in combination with bortezomib, melphalan, and prednisolone, on the basis of the ALCYONE trial that reported an 18-months PFS of 72% in the daratumumab group versus 50% in the control arm [14], as well as for the treatment of RRMM [12,13,15]. In the POLLUX trial investigating lenalidomide and dexamethasone with or without daratumumab, and the CASTOR trial investigating bortezomib and dexamethasone with or without daratumumab, the addition of daratumumab resulted in a considerable improvement of ORR (92.9% versus 76.4%, *p* < 0.001, and 82.9% versus 63.2%, *p* < 0.001, respectively). Consequently, daratumumab has become a standard of care in multiple myeloma [12,13]. We have recently reported on our experience with the daratumumab containing five-drug combination therapy “Pom-PAD-Dara” (pomalidomide, bortezomib, doxorubicin, dexamethasone, and daratumumab). Our results demonstrated an ORR of 78% in heavily pretreated patients with RRMM with a manageable safety profile, and patients with penta-refractory MM can also benefit from this treatment regimen [16]. mAbs-based treatment strategies represent a new dimension of precision medicine in the treatment of MM. However, as mAbs are increasingly being used, even for frontline/early treatment lines, the problem of resistance to mAbs emerges and motivates the development of novel immunotherapeutic agents to provide daratumumab and elotuzumab refractory patients with alternative treatment options. In the last few years, diverse mAbs-derivatives, e.g., the so-called bispecific antibody (bsAb), immunoconjugates, and chimeric antigen receptor-modified (CAR)-T-cells, have been developed for patients with RRMM and are currently under clinical investigation [17]. The three above-mentioned novel immunotherapeutic strategies lead to a revolution of anti-myeloma treatment, especially for end-stage RRMM, and might provide a new chance for these patients.

In this review, we provide an overview of the rationale, current clinical development, resistance mechanisms, and future directions of bsAbs in MM that have, together with CAR-T-cell therapies and immunoconjugates, the potential to pave the way towards a novel era of treatment for MM.

## 2. Rationale and Potential Targets of bsAbs in MM

### 2.1. Rationale and Biological Design of bsAbs

T-cells play a crucial role in the adaptive immune response to tumor cells through a variety of immune functions, including antigen recognition, cytokine production, immune regulation, induction of cellular lysis, and tumor cell elimination [18]. Therefore, using T-cells as a “weapon against tumor cells” has been considered as a therapeutic strategy for patients with MM.

Although experience with allogeneic SCT in MM is generally limited and the current guidelines do not recommend upfront allogeneic SCT, particularly outside clinical trials, allogeneic SCT has shown some efficacy and has improved long-term survival, especially in high-risk MM [4,5,6]. Moreover, donor lymphocyte infusions (DLI) has been reported to be effective in RRMM because of the graft-versus-myeloma effect [19,20,21,22,23,24], which underlined the anti-tumor function of donor T-cells in RRMM. Thus, recruiting and harnessing the patients’ own T-cells to eliminate MM cells might also be a promising immunotherapeutic strategy, notably, without the risk of graft-versus-host disease (GvHD). Utilizing bsAbs, MM cells can be directly linked with the T-cells of the patients, and, in this way, diverse anti-tumor cytotoxic mechanisms can be activated. Supported by this rationale, bsAbs have been developed for MM.

There are various technologies to build a bsAb, as discussed in a previous review article [25]. In general, the bsAbs developed for MM can be divided into two main groups, namely: bispecific T-cell engagers (BiTE^®^ (Amgen, Thousand Oaks, CA, USA)) and DuoBody^®^ (Genmab A/S, Copenhagen, Denmark) [26]. While a BiTE molecule consists of two different single chain variable fragments (scFv), a DuoBody is a mAb with two different antigen-binding fragments (Fab) and a functional constant region fragment (Fc) [27]. The presence of the Fc domain can promote the stability of the molecule and increase the half-life time [28].

### 2.2. Potential Targets of bsAbs for MM: From Preclinical to Clinical Trials

To facilitate an immune synapse, bsAbs have to be able to recognize MM cells and activate T-cells [26]. CD3 is a T-cell co-receptor, and the CD3-T-cell receptor (TCR) complex plays a crucial role in T-cell activation [29]. Thus, CD3 is considered a suitable target of bsAbs to engage T-cells. Another binding moiety of bsAbs is required for the recognition of MM cells. So far, several immune targets, including B-cell maturation antigen (BCMA), CD38, CD19, G-protein coupled receptor family C group 5 member D (GPRC5D), and Fc receptor-homolog 5 (FcRH5), have been considered suitable for bsAbs in MM [17]. Currently, bsAbs targeting the above-mentioned antigens are under clinical investigation. Here, we will provide an overview of these target antigens and the preclinical data of bsAbs in MM.

BCMA, a membrane protein also known as tumor necrosis factor receptor superfamily 17 (TNFRSF17), has been reported to be highly expressed by mature B-cells, including MM cells, and can promote myeloma cell growth in humans [30,31]. In addition, BCMA is almost absent in other cell lineages such as hematopoietic stem cells and normal human tissues. Because of these characteristics, BCMA has been considered a promising immune target for the therapy of MM [30]. In the preclinical setting, BI 836909, a BCMA/CD3 BiTE, induced T-cell activation, the release of cytokines, and the selective lysis of BCMA-positive myeloma cells [32].

CD38 is a type II transmembrane glycoprotein with an ectoenzyatic activity that also regulates cell adhesion, signaling events, and intracellular calcium mobilization [33]. The CD38 expression level is increased in myeloma cells [34,35]. Therefore, CD38-targeted mAb, such as daratumumab, has been developed for MM. Recently, Zuch de Zafra et al. reported on the considerable anti-myeloma activity of AMG424, a novel CD38/CD3 BiTE, without triggering excessive cytokine release [36]. However, CD38 has also been reported to be expressed in non-hematopoietic tissues, e.g., the gastrointestinal tract or cerebellar Purkinje cells [37], which is associated with on-target off-tumor toxicity [38].

The CD19/CD3 BiTE blinatumomab has shown a remarkable anti-tumor efficacy in CD19 positive cells [39], and was approved by the FDA for the treatment of B-cell precursor acute lymphoblastic leukemia (B-ALL) in 2014 [40]. In B-cell lineage, CD19 is widely expressed. Even if only approximately 10% of MM cells are evaluated CD19 positive by conventional flow cytometry [41], a higher proportion expresses CD19 on low levels, which can only be revealed by high-resolution imaging techniques [42]. This provides a rationale for targeting CD19 with highly potent directed immunotherapies, such as bsAbs or CAR-T-cells.

GPRC5D is a novel surface receptor on myeloma cells, whose overexpression is associated with a poor prognosis of patients [43]. In in vitro and in murine models, the GPRC5D/CD3 DuoBody JNJ-64407564 can induce the T-cell mediated killing of GPRC5D positive plasma cells [44]. These findings provide a proof of concept for targeting GPRC5D with bsAb in MM.

**Figure 1:**
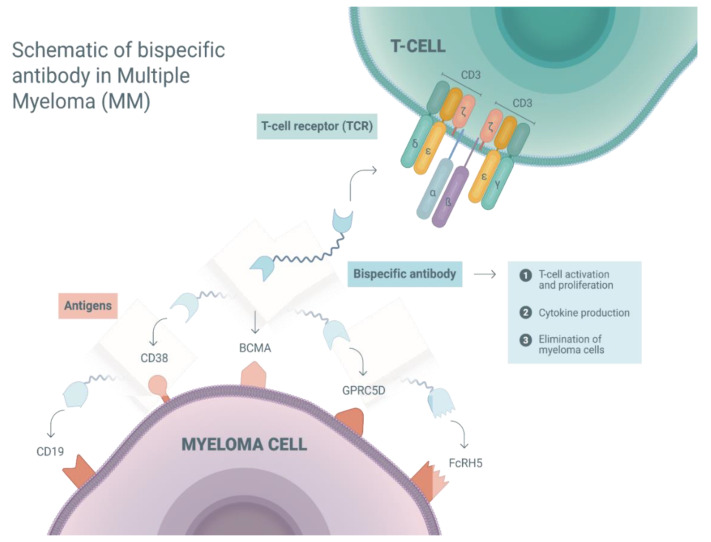
Schematic of a bispecific antibody in multiple myeloma (MM): mechanisms of action and potential targets. Bispecific antibodies facilitate an immune synapse between T-cell and myeloma cell via recognizing and binding the surface antigens on both cells. In this way, T-cells are activated, and this leads to a variety of immune reactions, such as T-cell proliferation, cytokine production, immune regulation, induction of cellular lysis, and tumor cell elimination. G-protein coupled receptor family C group 5 member D (GPRC5D); Fc receptor-homolog 5 (FcRH5); B-cell maturation antigen (BCMA).

FcRH5, also referred to as FcRL5, CD307, or IRTA2 (Immunoglobulin Superfamily Receptor Translocation Associated 2), is a membrane protein of an unknown function, which is exclusively expressed in B-cell lineage, including myeloma cells [45,46]. In preclinical studies, in vitro and in vivo data suggest that FcRH5/CD3 bsAbs can successfully activate T-cells, induce cytokine production, and eliminate malignant plasma cells [47,48]. Taken together, the targets for bsAbs are specific surface antigens on myeloma cells. Preclinical studies have demonstrated the anti-myeloma activity of bsAbs in in vitro or in animal models, and provided a rationale for clinical investigation. We summarized the mechanisms of action and the potential targets of BsAbs for MM in Figure 1. To date, a variety of clinical trials has been registered to investigate bsAbs, which target the five above mentioned antigens.

In addition, SLAMF7 (CS1 or CD319), a member of the signaling lymphocytic activation molecule family, and CD138 (syndecan-1), a heparan sulfate proteoglycan that acts as a co-receptor for growth factors and chemokines, are also highly expressed on malignant plasma cells [49,50]. To date, bsAbs targeting SLAMF7 and CD138 have been evaluated in preclinical studies, and have shown a promising efficacy in cell lines or animal models [50,51,52]. However, the development of SLAMF7-and CD138-directed bsAbs is still in a very early phase, and there is currently no clinical trial investigating these agents.

## 3. Clinical Development of bsAbs for MM

The first approved bsAb was blinatumomab for RR B-ALL. In a recent phase three study, blinatumomab showed significantly longer overall survival (OS) and significantly higher complete remission (CR) rates compared with the chemotherapy group (median OS: 7.7 months versus 4.0 months, *p* = 0.01; CR rates: 44% versus 25%, *p* < 0.001) [53]. As mentioned above, preclinical studies demonstrated the efficacy of bsAbs for MM in in vitro or in animal models. Here, we will review the clinical trials investigating bsAbs in MM.

### 3.1. bsAbs Targeting BCMA

The BCMA/CD3 BiTE AMG420, also known as BI 836909, is the first-in-class bsAb in MM [32]. In a phase 1 dose escalation study (NCT02514239), 42 RRMM patients without extramedullary disease (EMD) received AMG420 at 0.2–800 μg/d as continuous infusion. The ORR was 31%. At the maximum tolerated dose (MTD) of 400 μg/d, seven out (70%) of ten patients achieved a partial remission (PR) or better, with minimal residual disease (MRD) negative CR in five (50%) patients. Adverse events (AEs) ≥ grade 3 included 19% (*n* = 8) infection, 5% (*n* = 2) peripheral polyneuropathy, 2% (*n* = 1) edema, and 2% (*n* = 1) cytokine release syndrome (CRS). In three (7%) patients, the duration of response was >1 year [54]. Currently, another phase-1/-2 study (NCT03836053) evaluating the recommended dose of AMG420 is ongoing. Altogether, early clinical data demonstrated the efficacy of AMG420 in RRMM. A major limitation of AMG420 is its short half-life time, which requires continuous infusion via a central venous access in order to achieve a steady therapeutic plasma level [17].

Several other BCMA/CD3 BiTEs or DuoBodies with an extended half-life time, such as AMG701, PF-06863135, CC-93269, JNJ-64007957, REGN-5458, REGN-5459, and TNB-383B, are presently undergoing early clinical investigation. Notably, some of the agents, e.g., JNJ-64007957 and CC-93269, can be administered as a subcutaneous injection—a further step towards the improved clinical application of these therapies. Recently, the clinical development of AMG420 has been interrupted to further proceed that of AMG701, which shows an extended half-life time and does not require continuous intravenous infusion.

PF-06863135 (PF-3135), another BCMA/CD3 bsAb for RRMM, which has a longer half-life time than AMG420, is currently under clinical investigation in a phase 1 dose escalation study (NCT03269136). So far, 17 patients have received PF-3135 as once weekly bolus infusion in six dose escalation groups. In general, PF-3135 was well tolerated, with three (18%) patients developing AEs ≥ grade 3 (elevated alanine aminotransferase/aspartate aminotransferase, leukocytopenia, neutropenia, and lymphopenia). As of April 2019, response data were available for 16 patients. Of these, one (6%), six (35%), and nine (53%) patients had a minor response (MR), stable disease (SD), and progressive disease (PD), respectively [55].

In the phase 1 multicenter trial (NCT03486067), as of May 2019, 19 patients received CC-93269 at a dose of 0.15–10 mg as once weekly intravenous infusion over two hours. Treatment-related AEs ≥ grade 3 included 53% (*n* = 10) neutropenia, 42% (*n* = 8) anemia, 26% (*n* = 5) infection, and 21% (*n* = 4) thrombocytopenia. One patient treated with CC-93269 at a dose of 10 mg died due to CRS. The response data of the 12 patients treated with ≥6 mg CC-93269 demonstrated an ORR of 83%, and nine (75%) patients achieved MRD-negative CR [56].

JNJ-64007957 (teclistamab) is currently under investigation within a phase 1 trial (NCT03145181). As of January 2020, 66 patients were treated with JNJ-64007957 at 0.3–270 µg/kg. Two (3%) patients suffered from neurotoxicity ≥ grade 3. Other AEs ≥ grade 3 were infection (21%, *n* = 14), neutropenia (20%, *n* = 13), anemia (14%, *n* = 9), delirium (2%, *n* = 1), and thrombocytopenia (2%, *n* = 1). One patient died due to pneumonia (grade 5). The response data showed an ORR of 38% (20/52), with 78% (7/9) patients responding in the group of the highest dose. Two patients reached MRD-negativity at 10^−6^ [57].

In another study (NCT04108195), the first clinical trial investigating bsAb as part of a multi-targeted combination therapy, JNJ-64007957 is given in combination with daratumumab, and the results are eagerly awaited. Clinical trials evaluating BCMA-directed bsAbs are summarized in Table 1.

### 3.2. bsAbs Targeting GPRC5D, CD38, FcRH5 and CD19

At present, JNJ-64407564 is the only GPRC5D/CD3 bsAb undergoing clinical investigation (NCT04108195 and NCT03399799). Of note, the study NCT04108195 aims to evaluate the combination therapies JNJ-64407564 plus daratumumab, and BCMA/CD3 bsAb JNJ-64007957 plus daratumumab. As mentioned above, this is the first bsAb-based multi-targeted combination therapy investigated in clinical trials (Table 1).

CD38/CD3 bsAb products GBR-1342 and AMG424 are presently under investigation in phase 1 trials (NCT03309111 and NCT03445663). In addition, FcRH5/CD3 targeted bsAb BFCR4350A is evaluated in the study NCT03275103. The clinical data of these trials are pending.

The CD19/CD3 bsAb blinatumomab has been approved for B-ALL. To date, there is only limited experience with blinatumomab in patients with MM. Pratz et al. reported on a patient with RRMM and secondary ALL who received two cycles of blinatumomab at a dose of 28 µg/d. This patient achieved a MRD negative CR of ALL and a very good partial remission (VGPR) of MM [58]. Moreover, a systematic investigation of blinatumomab for the treatment of RRMM patients was intended within a pilot study (NCT03173430). However, this study was terminated prematurely due to slow accrual.

### 3.3. Safety Profile of bsAbs in MM

As shown in Table 1, clinical experience with bsAbs in MM is still very limited. The current available data on the safety profile of bsAbs in MM are based on preliminary results from the four phase 1 studies investigating BCMA-directed bsAbs (NCT02514239, NCT03269136, NCT03486067, and NCT03145181). Overall, AMG420, PF-3135, CC-93269, and JNJ-64007957 were well tolerated, with infection and blood count changes being the most common AEs ≥grade 3, which were generally manageable. Of note, one patient who received JNJ-64007957 within the clinical trial NCT03145181 died due to pneumonia, which, however, was classified as unrelated to treatment by the investigator [57]. Moreover, CRS ≥grade 3 was reported in patients treated with AMG420 [54] and CC-93269 [56], and CRS also resulted in a toxic death in the study of CC-93269 (NCT03486067) [56]. To date, the safety data of bsAbs targeting GPRC5D, CD38, FcRH5, and CD19 are still pending. Altogether, most of the bsAbs-related AEs are manageable. However, severe CRS could also be life threating, and the safety of bsAbs in MM should be further evaluated.

## 4. Resistance Mechanisms

So far, only two bsAbs have obtained regulatory approval as cancer drugs. Catumaxumab, the first-in-class bsAb, targeting epithelial cell adhesion molecule (EpCAM) and CD3, was approved by the European Medicines Agency (EMA) in 2009 for the treatment of malignant ascites, however, due to financial reasons, the marketing authorization was withdrawn voluntarily after less than a decade [59]. Therefore, most of the insights into resistance mechanisms to the treatment with bsAbs come from the second approved bsAb, blinatumomab, or agents currently tested in phase-1/-2 clinical trials. Mechanistically, reasons for relapses or failure to respond to therapy with bsAbs include immune and tumor cell intrinsic factors.

The restriction of T-cell functionality can result from different reasons. One thoroughly studied effect is the induction of immune checkpoint expression due to the strong T-cell activation conferred by the bsAb [60,61]. The release of proinflammatory cytokines, such as interferon γ [62], can trigger the increase of programmed death-ligand 1 (PD-L1) expression on the tumor cells that, in turn, can induce T-cell anergy and enable tumor escape [63]. The disruption of the PD-1/PD-L1 axis has resulted in improved tumor cell elimination, T-cell proliferation, and cytokine production in vitro [64]. Checkpoint signaling is an important immunosuppressive component of the myeloma microenvironment [65] and, indeed, in preclinical investigations, the anti-myeloma activity of the FcRH5/CD3 bsAb was limited by PD-1/PD-L1 signaling, however, the PD-L1 blockade significantly enhanced myeloma cell elimination in vitro [48]. Currently, various clinical studies are investigating the concept of bsAbs/checkpoint inhibition combination therapies (NCT02879695, NCT03340766, and NCT03160079), and promising first safety data have been reported for blinatumumab in combination with pembrolizumab in patients with RR B-cell neoplasias [66]. Other factors that drive T-cell anergy in the myeloma microenvironment are immunosuppressive cell subsets like regulatory T-cells (Tregs) [67,68] and myeloid-derived suppressor cells (MDSCs) [69], or cytokines like interleukin-10 (IL-10) and transforming growth factor-β (TGF-β) [70]. In fact, increased levels of Tregs were associated with poor response to blinatumumab in B-ALL patients [71]. Therefore, comparable resistance mechanisms mediated by the immunosuppressive tumor microenvironment are likely in MM. In addition, other than CAR-T-cells, bsAbs that are currently available for MM patients do not have a co-stimulatory domain, which might also influence the efficacy of these agents [25,72,73,74]. A recent study suggested that CD28 co-stimulation might enhance the efficacy of CD19/CD3 bsAb in vitro [73]. This finding highlighted the importance of co-stimulatory signals to the T-cells, and opens up another strategy for how to improve bsAb efficacy. Currently, the patients with the most unmet clinical needs are patients with RRMM that often feature aggressive disease characteristics and have had numerous courses of previous therapy. In these patients, MM-related immune dysfunction can be aggravated by therapy associated numeric and functional impairment the T-cell compartment [75,76]. Therefore, the successful further development of bsAb-based therapy will also have to take these factors into account.

The leading tumor cell intrinsic factor that limits the efficacy of bsAbs is related to the expression changes of the target antigen. For CD19-directed, T-cell-based immunotherapies, CD19 negative relapses have been observed in 7–25% of patients treated with chimeric antigen receptor (CAR)-T-cells [77,78,79], and in approximately 10% of patients treated with blinatumomab [80,81]. Genetic alterations, splice variants [82], myeloid lineage shift [83], and the disruption of CD19 membrane trafficking [84] have been identified as mechanisms resulting in the loss of CD19 surface positivity. In MM, BCMA-directed CAR-T-cell therapy resulted in a reduction of BCMA expression levels [85], and cases of BCMA, negative relapses have been reported [86]. So far, robust data on target antigen expression at relapse after bsAb therapy in MM are still missing [54]. However, the heterogeneous nature of the disease, characterized by a mixture of a subclones competing for predominance spontaneously or under therapeutic pressure predisposes for antigen escape variants [87]. Multi-targeted immunotherapeutic strategies, e.g., the combination of anti-CD38 antibody daratumumab with anti-BCMA or anti-GPRC5D bsAb (NCT04108195), might be a promising approach to tackle this challenge.

## 5. Discussion and Future Directions

The first bsAb was reported by Staerz and Bevan in 1985 [88]. With blinatumomab obtaining the FDA approval for the treatment of B-ALL in 2014, this novel immunotherapeutic strategy utilizing the patient’s own T-cells has become an attractive option for hematological malignancies [89]. In the last few years, bsAbs evaluated in early-phase clinical trials have also been available for heavily pretreated late-stage RRMM patients. Moreover, in contrast to CAR-T-cell therapy, bsAbs are an off-the-shelf product that can be given to the patients instantly, without the ex vivo processing of the patients’ T-cells [26]. However, CAR-T-cell therapy has shown a higher ORR compared with bsAb, as demonstrated in studies investigating BCMA-directed bsAb and CAR-T-cells in RRMM, as well as CD19-directed agents in B-ALL [53,54,90,91,92,93]. As previously mentioned, in contrast to CAR-T-cells, a lack of co-stimulation might induce T-cell anergy and compromise the efficacy of bsAbs. Furthermore, a study of blinatumomab in B-ALL also showed a response to retreatment at relapse (ORR 36%) after initial treatment with blinatumomab [94]. To date, clinical trials evaluating CAR-T-cell retreatment are still ongoing.

So far, bsAbs have been tested in patients with RRMM within clinical trials, and the currently available data demonstrate the anti-myeloma efficacy of bsAbs. However, there is still no published experience of bsAb in patients with newly diagnosed (ND) MM. As novel immunotherapeutic agents, e.g., daratumumab, were approved by the FDA for NDMM as first-line therapy in 2019, studies investigating the potential role of bsAbs in earlier lines of therapy for MM are also warranted.

Another challenge for the successful implementation of bsAbs into existing treatment paradigms is to improve efficacy and to overcome drug resistance to single-agent therapies. As mentioned above, multi-targeted treatment approaches using a combination of two or more immunotherapeutic agents might be a promising strategy that is currently under clinical investigation. In addition, bsAbs targeting other antigens, such as CD138 and SLAMF7, have also been explored in preclinical studies using cell lines or animal models [50,51,52], and might provide further options for patients with MM. However, awareness of antigen escape, due to tumor heterogeneity and selection pressure, especially under highly effective immunotherapies, is also an important point that needs to be taken into account in the management of patients suffering from MM. Moreover, the on-target off-tumor effect should be considered, especially when targeting antigens that are also expressed in non-hematopoietic tissues, e.g., CD38.

For MM, bsAbs and trispecific antibodies (tsAbs) engaging natural killer (NK) cells have also been explored in preclinical studies. Ross et al. reported the NK-cell mediated lysis of BCMA-positive myeloma cell lines induced by AFM26, a BCMA/CD16A directed bsAb [95]. Moreover, a BCMA/CD200/CD16A tsAb was used by Gantke et al. to link NK-cells and myeloma cells, and BCMA and CD200 double-positive myeloma cells showed increased cellular lysis compared with single-positive cells [96]. Furthermore, Banaszek et al. reported on hemibodies consisting of two different antigen-specific scFvs fused to the variable light or variable heavy chain domains of a CD3 antibody. If both hemibodies simultaneously bind their respective antigens on a single cell, a T-cell engaging CD3 binding domain is generated [97]. In preclinical models, hemibodies can preferentially induce the cellular lysis of double-positive cells and spare single-positive cells [97]. Taken together, these encouraging findings support the rationale of NK-cell or hemibody-based anti-myeloma treatment, which could further widen the spectrum of antibody-based therapy in MM.

A combination of novel immunotherapeutic agents, such as bsAbs or CAR-T-cells, and established therapies, like autologous SCT or daratumumab, might provide a new therapy strategy for high-risk MM or daratumumab refractory patients. Moreover, the immunomodulatory effects of daratumumab might also act synergistically with these novel immunotherapeutic agents. With the ongoing drug development, there will be more and more therapeutic options available for patients with RRMM and high-risk MM.

## 6. Conclusions

In summary, bsAbs have shown a promising efficacy in preclinical and early clinical studies in MM. Further clinical investigation of bsAbs for the treatment of MM is ongoing. Even if definitive data are still very limited, the use of bsAbs for the treatment of MM appears to be a promising new dimension of precision medicine.

## Figures and Tables

**Table 1:** Overview of clinical trials investigating bispecific antibodies (bsAbs) in multiple myeloma.

ClinicalTrials.gov Identifier	Bispecific Antibody	Targets	Study Phase	Estimated Enrollment	Current Status *	Response Rates	AEs ≥ Grade 3
NCT02514239	AMG420	BCMA/CD3	1	43 patients	Active, not recruiting	42 patients were treated. ORR: 31% (13/42); at MTD of 400 µg/d: 70% (7/10) including 50% (5/10) MRD-negative CR	19% (*n* = 8) infection, 5% (*n* = 2) peripheral polyneuropathy, 2% (*n* = 1) edema, and 2% (*n* = 1) CRS
NCT03836053	AMG420	BCMA/CD3	1/2	15 patients	Recruiting	N/A	N/A
NCT03269136	PF-3135	BCMA/CD3	1	80 patients	Recruiting	17 patients were treated. Response data were available in 16 patients. CBR: 41%; 6% (1/16) MR, 35% (6/16) SD, and 53% (9/16) PD	18% (*n* = 3) including elevated transaminase, leukocytopenia, neutropenia, and lymphopenia
NCT03145181	JNJ-64007957	BCMA/CD3	1	160 patients	Recruiting	Activity was observed in 52 patients who received ≥ 38.4 µg/kg. ORR: 38% (20/52); at 270 µg/kg: 78% (7/9)	21% (*n* = 14) infection, 20% (*n* = 13) neutropenia, 14% (*n* = 9) anemia, 3% (*n* = 2) neurotoxicity, 2% (*n* = 1) delirium, and 2% (*n* = 1) thrombocytopenia. One patient died due to pneumonia (grade 5)
NCT03933735	TNB-383B	BCMA/CD3	1	72 patients	Recruiting	N/A	N/A
NCT03486067	CC-93269	BCMA/CD3	1	120 patients	Active, not recruiting	12 patients were treated with ≥ 6 mg CC-93269. ORR: 83% (10/12) with 58% (7/12) ≥ VGPR and 33% (4/12) sCR; MRD-negative CR rate: 75% (9/12)	53% (*n* = 10) neutropenia, 42% (*n* = 8) anemia, 26% (*n* = 5) infection, and 21% (*n* = 4) thrombocytopenia. One patient died due to CRS (grade 5)
NCT03287908	AMG-701	BCMA/CD3	1	270 patients	Recruiting	N/A	N/A
NCT03761108	REGN-5458	BCMA/CD3	1/2	74 patients	Recruiting	N/A	N/A
NCT04083534	REGN-5459	BCMA/CD3	1/2	56 patients	Recruiting	N/A	N/A
NCT04108195	JNJ-64007957 JNJ-64407564	BCMA/CD3 GPRC5D/CD3	1	100 patients	Recruiting	N/A	N/A
NCT03399799	JNJ-64407564	GPRC5D/CD3	1	185 patients	Recruiting	N/A	N/A
NCT03309111	GBR-1342	CD38/CD3	1	125 patients	Recruiting	N/A	N/A
NCT03445663	AMG424	CD38/CD3	1	120 patients	Recruiting	N/A	N/A
NCT03275103	BFCR4350A	FcRH5/CD3	1	130 patients	Recruiting	N/A	N/A
NCT03173430	Blinatumomab	CD19/CD3	1	6 patients **	Terminated	N/A	N/A

AE—adverse event; BCMA—B-cell maturation antigen; CBR—clinical benefit rate; CR—complete remission; CRS—cytokine release syndrome; FcRH5—Fc receptor-homolog 5; GPRC5D—G-protein coupled receptor family C group 5 member D; MR—minor response; MRD—minimal residual disease; MTD—maximum tolerated dose; N/A—not available; ORR—overall response rate; PD—progressive disease; sCR—stringent complete response; SD—stable disease; * as of 1 May 2020; ** actual enrollment.
